# Gastrointestinal Stromal Tumors: A Review of Case Reports, Diagnosis, Treatment, and Future Directions

**DOI:** 10.5402/2012/595968

**Published:** 2012-04-12

**Authors:** Christopher B. Tan, Wanqing Zhi, Ghulamullah Shahzad, Paul Mustacchia

**Affiliations:** ^1^Department of Internal Medicine, Nassau University Medical Center, East Meadow, NY 11554, USA; ^2^Department of Gastroenterology, Nassau University Medical Center, East Meadow, NY 11554, USA

## Abstract

Gastrointestinal stromal tumor (GIST) is a nonepithelial, mesenchymal tumor first described by Mazur and Clark in 1983. Since then, its molecular biology has been studied in great detail. Special interest in the role of tyrosine kinase in its regulation has been the target by different drug research. Mutation in *c*-*kit* exons 9, 11, 13, 17 and PDGFRA mutation in exons 12, 14, 18 are responsible for activation of gene signaling system resulting in uncontrolled phosphorylation and tissue growth. However, 5 to 15% of GISTs does not harbor these mutations, which raises additional questions in another alternate signaling pathway mutation yet to be discovered. Diagnosis of GISTs relies heavily on KIT/CD117 immunohistochemical staining, which can detect most GISTs except for a few 3% to 5% that harbors PDGFRA mutation. Newer staining against PKC theta and DOG-1 genes showed promising results but are not readily available. Clinical manifestation of GISTs is broad and highly dependent on tumor size. Surgery still remains the first-line treatment for GISTs. The advancement of molecular biology has revolutionized the availability of newer drugs, Imatinib and Sunitinib. Together with its advancement is the occurrence of Imatinib/Sunitinib drug resistance. With this, newer monoclonal antibody drugs are being developed and are undergoing clinical trials to hopefully improve survival in patients with GISTs.

## 1. Introduction

Gastrointestinal stromal tumor (GIST) has an estimated annual incidence in the US of approximately 3,000–4,000, making it the most common primary mesenchymal tumors of the gastrointestinal tract [1, 2]. GISTs are thought to arise from interstitial cells of Cajal (ICC) or their stem cell precursors which are normally part of the autonomic nervous system of the intestine [2, 3]. ICC serves as a pacemaker function in controlling motility. GISTs usually arise in the stomach in 40% to 70%, in the small intestine in 20% to 40%, and less than 10% in the esophagus, colon, and rectum [3–6]. GISTs can develop outside the intestinal tract, within the abdominopelvic cavity such as the omentum, mesentery, uterus, and the retroperitoneum; they are called extragastrointestinal stromal tumors (eGIST), usually behaving aggressively [2, 4, 7, 8]. GIST has been shown to affect men (55%) more than women with median age of 55–60 [9].

In 1941, Golden and Stout described a set of mesenchymal tumors arising in the bowels which were mistakenly identified as tumors arising from the smooth muscle's cells as leiomyoblastoma, leiomyoma, and leiomyosarcoma [2]. Although the term “GIST” was first used in 1983 by Mazur and Clark, it was in 1998 when Japanese researchers discovered the presence of a KIT protein and the possibility of *kit* mutations, which distinguishes GISTs from other similar tumors. Prior to that time, KIT testing by immunohistochemistry was not readily available and GIST was not always clearly recognized as a distinct sarcoma type [2].

Since the discovery of KIT protein, its expression in GIST has been a great area of molecular biologic research. It revolutionized its pathophysiology and relationship in the development of stromal tumors. Estimated 85% of GIST tumors were found to have an active mutation in the *kit* proto-oncogene while only 3–5% mutation in PDGFRA [1].

For many years, the mainstay of treatment for GIST is surgical resection. Unfortunately, the results of surgery alone have been inadequate, with up to 50% of patients developing tumor recurrence within the first five years [1]. Postoperative chemotherapy with conventional agents and radiation therapy were ineffective as well [1, 4]. With the recent advancement of proto-oncogene testing and immunohistochemical staining, treatment for GIST has evolved with therapies directed against specific *kit*/PDGFRA proto-oncogene, showing promising results. The use of small-molecule kinase inhibitors that target the underlying pathogenic mutant kinase has revolutionized the treatment of GIST. However, recently reported cases are showing emergence of drug-resistant tumor clones, which limit the long-term benefits of these drugs [10].

This paper will summarize recent case reports, progress in the diagnosis and treatment of GIST, and how to approach patients with GIST as well as future directions in management of GISTs. The selection of case report was done at random, based on keywords “case reports in GIST,” “gastrointestinal stromal tumors case reports,” “extraintestinal GIST,” and “eGIST” using the search engine of pubmed, google scholar, and the directory of open access journals. The cases presented are only a representative of the numerous case reports regarding GISTs.

## 2. Molecular Biology

### 2.1. *c*-*kit*

**Figure 1:**
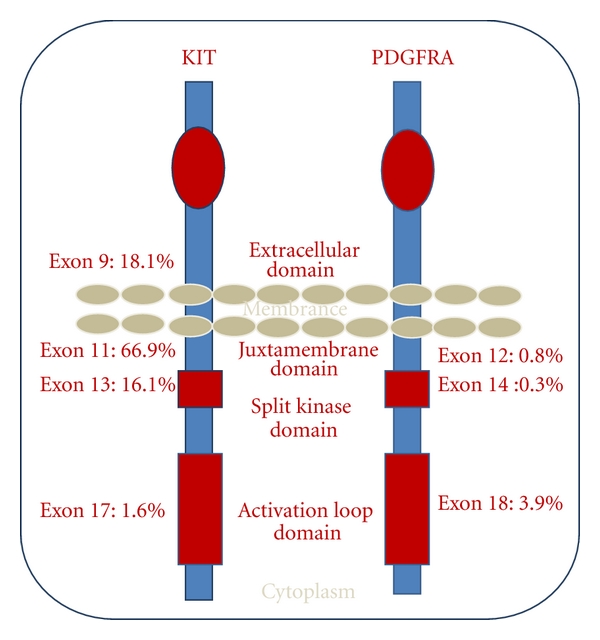
Schematic representation of KIT and platelet-derived growth factor receptor alpha (PDGFRA) molecule with location and frequency of mutation.

GISTs are mesenchymal tumors of the gastrointestinal tract characterized by their genetic expression of *kit* and immunohistochemical staining of CD117, which occurs in 85% to 95% of all GISTs [2, 10]. *kit* is a 145 kD transmembrane tyrosine kinase which serves as a receptor for stem cell factor [10]. The binding of stem cell receptor to *kit* results in homodimerization of its receptor with the activation of tyrosine kinase and concomitant activation of downstream intracellular signal transduction pathways, most notably RAS-RAF-MAPK and P13K-AKT-mTOR pathways [10]. This results in modification of several cellular functions, which includes adhesion, migration, differentiation, and cellular proliferation with decrease in cellular apoptosis. These oncogenic potentials would ultimately lead to neoplasia [2]. The mutation of the *kit* proto-oncogene tends to cluster in four exons, namely, exon 9 (extracellular domain), exon 11 (intracellular juxtamembrane domain), exon 13 (split kinase domain), and exon 17 (kinase activation loop), (Figure 1) [2, 3].

Exon 11 mutations, which encode for juxtamembrane domain, are the most common mutated regions of *kit*. They account for 70% of all the tumors and do not appear to be associated with any specific location, size, or clinical outcome [2]. In-frame deletions of 1 or more codons in exon 11 *kit* are the most common mutations, accounting for 60% to 70%. The majority of these mutations involves the proximal part of *kit *exon 11 between codons Gln550 and Glu561 [3, 11]. Deletion of Trp557 and Lys558 in exon 11 codon, which is the most common simple deletion in GISTs, is associated with poorer clinical outcome with more aggressive metastatic behavior [3, 12].

Missense point mutation in *kit* exon 11 is the next most common type of mutation, occurring in 20% to 30% of GISTs. They involve almost exclusively three codons, Trp557, Val559, and Val560, in the proximal part, and Leu576 in the distal part of exon 11. GIST with missense mutation at these regions seems to have better prognosis in gastric but not in small intestinal tumors [3].

Exon 9 mutations are the second most often involved region which entails mutations of the extracellular domain [3, 10]. These account for 10% of tumors and are most commonly associated with GIST of the small bowel with a known aggressive clinical behavior [2, 3]. Nearly all mutations in exon 9 have been identical with 6-nucleotide duplications, encoding Ala502-Tyr503, this was initially reported by Miettinen and Lasota [3], Lux et al. [13].

Primary mutation of exon 13 (split kinase domain) and exon 17 (loop) are rare, accounting for <1% of the cases. Exon13 involves missense mutations resulting in substitution of Glu for Lys with a more malignant potential [3].

### 2.2. PDGFR Alpha

A closely homologous tyrosine kinase PDGFRA is seen in 5% to 7% of GISTs. They harbor mutations in decreasing order of frequency, involving exons 12, 14, and 18 (Figure 1) [3, 14–16]. *kit *and PDGFRA are mutually exclusive, and like *c-kit* they activate similar transduction pathways that support GIST oncogenesis but act at a different receptor site. Most PDGFRA mutant GISTs are located in the stomach, behaving aggressively. They have an epithelioid morphology with weak or negative immunohistochemical reaction to CD117 [2]. A case report by Todoroki et al. reports a PDGFRA mutation at exon 12, located at the greater omentum of the stomach with immunohistochemical staining that is weakly positive for CD117, showing an epithelioid morphology (Table 1). The patient responded to Imatinib treatment with no recurrence after six months. 

More than 80% of PDGFRA mutations occur in exon 18. They are mostly missense mutations leading to substitution of Asp to Val. These tumors are usually resistant to treatment with imatinib [3]. Missense mutation affecting exon 14 has also been reported with substitution of Asn to Lys or Tyr. These tumors have better prognosis than the earlier [3, 16]. On the other hand, mutations of exon 12 are extremely rare [3].

### 2.3. Wild Type

5% to 15% of GISTs do not harbor either *kit* or PDGFRA mutations and are known as wild-type GISTs [2, 5]. These tumors can be positive for CD117 and can be mistakenly labeled as an Imitanib-susceptible GIST [2, 9]. However, these tumors are considered less responsive to imatinib treatment with a poorer prognosis. It has been suggested that these tumors harbor the insulin growth factor 1 receptor (IGF1R) mutation, which is highly expressed in both adult and pediatric wild-type GIST. The downregulation of IGF1R activity would lead to cytotoxicity or induced apoptosis in experimental studies [5].

## 3. Clinical Features

The spectrum of clinical presentation in GIST is broad. It is largely dependent on tumor size and location. GIST-causing symptoms are usually larger in size, more than 6 cm in diameter [9]. The most common presentation of GIST is abdominal pain and/or GI bleeding [2, 42]. This may be acute, as in melena, hematemesis, or chronic insidious bleeding leading to anemia [2]. GIST can also cause symptoms secondary to mass effect, including satiety, bloating, and abdominal pain [2, 9]. In our case review, abdominal pain is the most common complaint, followed by mass effects and GI bleed. Other symptoms observed in our review include pelvic pain, pleuritic chest pain, small bowel obstruction, dysuria, altered bowel movement, nausea, and weight loss (Table 1).

About 70% of patients with GISTs develop symptoms; the remaining 20% to 30% are diagnosed incidentally or at autopsy [2, 9]. These findings correlate closely with our observation that 5 out of 32 (15%) case reports on GISTs were found incidentally [7, 20, 22, 27, 37]. Approximately 20% to 25% of gastric and 40% to 50% of small intestinal GISTs are clinically malignant. The most common metastatic sites include the abdominal cavity, liver, and rarely bones and soft tissues. GISTs very rarely, if not, metastasize to the lymph nodes and the skin [9, 43]. In the case reports that we reviewed, abdominal cavity was the most common metastatic site followed by the liver and the pancreas. No lymph node metastases were noted (Table 1).

### 3.1. Familial GISTs and GISTs Syndromes

Less than 5% of GISTs can be associated with one of the four tumor syndromes: familial GISTs, neurofibromatosis type 1 (NF1), Carney's triad (CT), and, recently, the Carney-Stratakis triad (CSS) [4, 44–46].

Familial GIST syndrome (FGS) has been reported and identified in different families worldwide. FGS is inherited as autosomal dominant pattern harboring multiple, sometimes diffuse GISTs. Clinical presentation of FGS includes hyperpigmentation, increase in the number of nevi, urticaria pigmentosa, and/or systemic mastocytosis [4, 44]. Dysphagia, which is physiologically different from true achalasia, has been reported in family members affected by FGS. Familial GIST syndrome usually presents with multiple GIST in the small bowel and to a lesser extent, in the stomach. It has also been described in the esophagus and the rectum [44]. Morphologically, these tumors are indistinguishable from sporadic GISTs and are characterized with low mitotic rates. Most of FGS also expresses CD117/KIT, as well as CD34 in immunohistochemical staining [4, 44].

Neurofibromatosis type I (NF1) can also harbor multiple GISTs in approximately 7% of patients. This results from germline mutation of NF-1 gene that encodes neurofibromin. They are often diagnosed in the late fifth and sixth decades of life with slight female predominance [4, 44]. The most characteristic findings of NF-1 include café au lait spots, axillary and inguinal freckling, multiple dermal neurofibromas, and Lisch nodules. Although gastrointestinal manifestations of NF-1 are less frequent than cutaneous manifestation, it is not uncommon. These symptoms include hyperplastic lesion of intestinal neural tissue, GISTs, endocrine cell tumor of duodenum, and the periampullary region, as well as other miscellaneous groups of tumors [47].

Clinical features of NF-1-associated GIST are more closely similar to CT than to CSS [44]. NF-1-related GISTs are usually multiple, occurring in the small bowel, exhibit a spindle-shaped morphology, and do not harbor either *kit *or PDGFRA mutations, although it can express KIT in immunohistochemical staining [4]. It is believed that the deficiency of neurofibromin promotes the growth of specific subtype of ICC in contrast to direct mutation of the *kit* signaling system seen in non-NF-1-GISTs [47]. Most cases of NF1-associated GIST have an indolent course, but some were mitotically active and were clinically malignant [48].

 The carney triad (CT) and the more recent Carney-Stratakis syndrome (CSS) are the two other syndromes that predispose to GISTs. CT was first described by Carney and colleagues in 1977. CT generally occurs in females at a younger age, typically before the age of 30, presenting with a combination of multiple gastric GIST, paraganglioma, and pulmonary chondroma [4, 44–46, 49]. These lesions tend to have higher risks of metastasis, particularly to the lymph nodes. They are morphologically different from sporadic GISTs. No germ-line mutation specific for CT has been discovered to date. Neither *kit* nor PDGFA proto-oncogene has been found on analysis of these patients [4, 44].

CSS occurs at a younger age group than that of CT, with mean age of 23 years old. Both males and females are equally affected [44]. CSS-related GISTs tend to be multiple, localized in the stomach, with an epithelioid morphology on biopsy. Clinically, these patients present with multifocal GISTs, paragangliomas, and pheochromocytomas. Carney Stratakis syndrome GISTs occur because of germline mutations in the enzyme succinate dehydrogenase (SDH) [4, 49]. In our review, four cases of NF-1-associated GIST were recorded (Table 1) [22, 37, 39, 40].

## 4. Pathologic Features

GISTs normally present a wide clinical-pathological spectrum, from a small incidental nodule to large pedunculated mass. They are usually described as a tan to white, well-circumscribed lesions within the walls of the stomach [4]. GISTs demonstrate either of the 3 main histologic cell types: spindle cell type (most common), epithelioid cell type, and the mixed spindle-epithelioid type [3–5, 9].

Spindle cell GISTs account for 70% of the tumors [48]. The same is the most commonly reported histological pattern on our review (Table 1). Histologic subtypes for spindle cell GISTs include sclerosing spindle-cell, palisading-vacuolated subtype, hypercellular subtype, and sarcomatous spindle cell [48].

Epithelioid cell's type accounts for the next 20% with the remaining showing mixed pattern. Epithelioid histological subtypes include sclerosing epithelioid variant, dyscohesive epithelioid, hypercellular epithelioid, and sarcomatous epithelioid GISTs. Epithelioid morphology is closely related to PDGFRA mutation with a more aggressive tumor behavior [48]. Todoroki et al. reported an epithelioid histological pattern in a GIST with PDGFRA mutation (Table 1) [34].

## 5. Immunohistochemical Staining

### 5.1. CD117/KIT

Greater than 95% of GISTs are positive for CD117/KIT but are no longer considered as an absolute requirement [3–5]. Commonly expressed but less GISTs-specific antigens are CD34, nestin, smooth muscle actin (SMA), caldesmon, calponin, vimentin, and embryonic smooth muscle myosin. GISTs are generally negative or are weakly positive for desmin. S100 positivity is rare but relatively more common in small intestinal GISTs than gastric GISTs [3, 4]. Tumors that can consistently test positive for KIT include mastocytoma, seminoma, pulmonary small cell carcinoma, and extramedullary myeloid tumors. Abdominal or GI tumors that may test positive for KIT are metastatic melanoma, clear-cell sarcoma (30% to 50%), Ewing's sarcoma (50%), childhood neuroblastoma (30%), angiosarcoma (50%), and some carcinoma [3].

### 5.2. CD34

CD34 is positive in 80% to 85% of gastric GISTs and about 50% in small intestinal GISTs. The spindle variants are more likely to stain with CD34 than the epithelioid variants. Sarcomatous variants have higher tendency to stain with CD34 than the nonsarcomatous histologic subtype [48]. Out of the 32 case reports reviewed, all were positive for CD117/KIT. One of these was weakly reactive to CD117/KIT that is related to PDGFRA mutation [34]; and another related to wild-type mutation [39]. 19 of these cases with spindle-shaped morphology were concomitantly positive for CD34. Other immune markers noted in the review include SMA, S-100, neuron-specific enolase (NSE) (Table 1).

### 5.3. Protein Kinase *C* Theta

Protein kinase C theta is a novel protein kinase, downstream effector in the *kit* signaling system that is involved in T-cell activation, signal transduction, and neuronal differentiation [48, 50, 51]. Various studies have shown that PKC theta is strongly expressed and is overexpressed in GISTs, but not in other sarcomas [51–53]. These studies established PKC theta as a diagnostic marker for GIST. Studies have also suggested that the loss of PKC theta expression could be responsible for inhibition of *kit* expression in GISTs, hence does not react to KIT staining [51]. In study conducted by kim et al. on 220 GIST tumors, 212 were positive to PKC theta (96%) while KIT was positive in 216 (98%). However, two samples that are PKC theta positive and KIT negative showed mutation in PDGFRA, indicating that PKC theta might be a useful marker in diagnosing KIT-negative PDGFRA mutation tumors [50]. Although, other investigators believe that PKC theta staining is often weak and less distinct than CD117/KIT staining [4].

### 5.4. DOG1

Discovered on GIST-1 (DOG-1) is a novel gene encoding for a hypothetical protein that has been ubiquitously expressed on GISTs [54, 55]. In a study conducted by West et al., immunoreactivity for DOG1 GIST samples was 97.8% reactive. They have demonstrated a reaction to DOG1 on tissues that express PDGFRA mutation that failed to react for KIT immunostaining. These tests were later confirmed with in situ hybridization for DOG1, *kit, *and PDGFRA mutation. DOG1 is highly expressed not only in typical GISTs but also in *kit*-mutation-negative GISTs [55]. Another study, conducted by Espinosa et al. on DOG1 antibody, showed a high sensitivity and specificity, with 87% immunoreaction to GISTs. In contrary, only 74% reacted to CD117/KIT immunostaining [54]. Since 5 to 7% of PDGFRA GISTs mutation and 5% of *kit*-mutated GISTs do not react to CD117/KIT, DOG-1 staining would be an essential tool for a more reliable diagnosis on GISTs [54, 55]. Moreover, PDGFRA GISTs mutation can still benefit from imatinib treatment, making DOG-1 an important tool in these conditions. DOG1 immunohistochemistry staining is commercially available in some countries, including the United States under the trade name Thermo Scientific, GenWay Biotech, LSBio, and Leica.

## 6. Risk Assessment in GIST

Tumor size, location, and mitotic index remain the main variables used in risk stratification systems first developed by the National Institute of health, the so-called Fletcher's criteria [4, 5]. Revised version of the NIH risk stratification system by inclusion of additional prognostic factors, such as nonradical resection (R1) and a tumor rupture that affects adverse outcomes, was proposed by several investigators; and was later referred to as the modified NIH criteria [56, 57]. Tumor location was subsequently shown to have independent prognostic value and was later incorporated into the Miettinen-Lasota/Armed Forces Institute of Pathology risk stratification system (AFIP) (Table 2) [58]. The AFIP system has the advantage of delivering numerically calculated risk of tumor relapse and/or progression, which is a vital tool in helping clinicians make solid therapeutic decisions [3, 56]. The guidelines have also been recommended by both the National Comprehensive Cancer Network (NCCN) and the College of American Pathologist (Table 2) [4]. The same guidelines were equally used by most of the case reports we have reviewed (Table 1). The major drawback of the AFIP system is its complexity, considering eight prognostic subgroups and further subdivision into different subgroups. This reduces the prognosis sensitivity and specificity of recurrence [56].

On the other hand, the NIH system has the tendency to overgrade gastric tumors and downgrade a subset of nongastric tumors as compared to the AFIP system [56]. The complexity of AFIP risk stratification led to the proposal of a TNM classification system for GISTs. The seventh edition of the international union against cancer (UICC) published on 2010 included, for the first time, a classification and staging system for GIST using the TNM system (Table 3, TNM system 2010) [59]. The principal aim of the TNM system is to facilitate a uniform and standardized analysis of malignant tumors based on their stage of development and degree of spread. Other investigators argued that using TNM system is no more than renaming the existing risk group that was developed by AFIP [56]. Whether TNM system is better than the current AFIP system in risk stratification needs to be further validated. None of the case reports we reviewed used the TNM system as a method of stratification (Table 1).

A recent population-based observational cohort study involving 2560 patients by Joensuu et al. compared the NIH criteria, the modified NIH criteria and the AFIP system for risk stratification for recurrence-free survival (RFS) in imatinib naive operable GISTs. Data from the study suggested that large tumor size, high mitotic count, nongastric location, presence of rupture, and male sex were the independent prognostic factors for RFS. The three criteria in the study did fairly accurate in estimating RFS with the modified NIH criteria, able to identify a single high-risk group. The group further concluded that most operable GISTs are cured with surgery alone in about 60% of cases, considering 15 years of RFS (95% CI, 56.2–63.6) and thus does not benefit from systemic adjuvant therapy [60]. The TNM system of risk stratification suggested by UICC was not included in this study.

## 7. Treatment

### 7.1. Surgery

Despite the impressive advances in targeted therapy, surgery resection with preservation of the pseudocapsule remains the primary mode of therapy for localized GISTs [4, 5, 61, 62]. Surgery is used in three main approaches, most commonly as an initial treatment (primary surgery) after diagnosis, especially if the tumor is solitary and can be easily removed. It can be used after neoadjuvant treatment to shrink the size of the tumor; and, in some cases, surgery is used for advanced metastatic disease for symptomatic relief, termed debulking surgery [62]. These tumors should be handled carefully to avoid tumor rupture and spread. Lymphadenectomy is not routinely recommended since GISTs, as mentioned before, rarely metastasize to the lymph nodes. GISTs respond poorly to conventional chemotherapy and radiation therapy [4].

**Figure 2:**
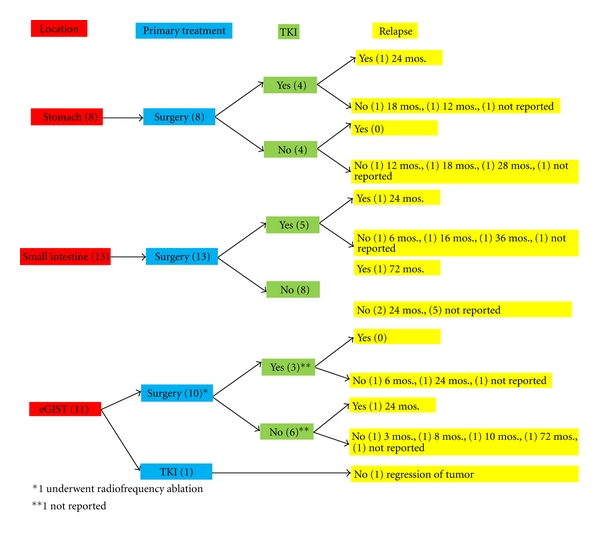
Summary of treatment options and relapse rate on 32 case reports.

**Figure 3:**
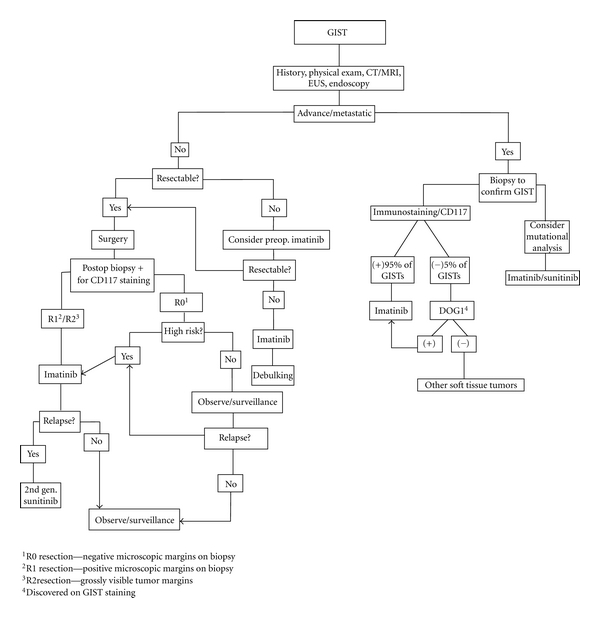
Schematic diagram on the stepwise approach to patients with GIST.

In our review of 32 case reports, 31 received operative treatment as the primary form of therapy. A case of a metastatic lesion by Dickhoff et al. did not receive surgical intervention; instead patient received Imatinib treatment with tumor regression on followup. This is in accordance with the NCCN guidelines for treatment of metastatic tumor (Figure 3) [5]. Furthermore, 18 out of 32 cases received surgery as the sole treatment with only two relapse cases after 24-month and 72-month followup (Table 1, Figure 2).

The 2010 National Comprehensive Cancer Network (NCCN) GIST Guidelines state that the first step in the management of a potentially resectable GIST is to determine its resectability with history/physical exam together with tests such as computed tomography (CT) and/or magnetic resonance imaging (MRI), chest imaging, endoscopic ultrasound (EUS), and endoscopy. PET scan is not routinely recommended [5]. If the mentioned test did not show any metastatic disease, preoperative biopsy of suspected GISTs is usually not indicated (Figure 3); the NCCN recommends a biopsy only if the tumor is unresectable, if the diagnosis in doubt, or if neoadjuvant therapy is planned [5, 63].

Before the imatinib era, resected GISTs can have high recurrence and failure rates with a 5-year survival of 28–35% [62, 63]. Tumors of more than 10 cm in size were associated with 5-year disease-free survival (DFS) of only 20% and median times to progression (TTP) of seven months to two years with only 10% of patients remained disease-free after followup [63]. Although a recent population-based observational cohort study by Joensuu et al. concluded that most patients with operable GISTs are cured by surgery alone with 60% estimated 15 years RFS, the study has a median tumor diameter of 5.5 cm with tumors mostly located in the stomach [60]. This raises additional questions as to the exact estimate of RFS, since the size and the location of the tumor have a prognostic implication in risk stratification.

### 7.2. Imatinib and Sunitinib

Imatinib mesylate and sunitinib maleate are competitive inhibitors of KIT and PDGFRA [4, 10]. Both drugs bind and stabilize the inactivated form of the receptor tyrosine kinases which leads to inhibition of phosphorylation and downstream KIT signaling activation. Its limited ability to bind to inactivated form of the tyrosine kinase is one of the reasons of drug resistance [10]. These drugs also differ on their binding targets. While Imatinib binds to a specific amino acid residue within the ATP binding pocket and the activation loop, Sunitinib interacts with a structurally different amino acid residue within the ATP binding pocket [4].

The usual starting dose of Imatinib is 400 mg per day. Large trials on low-dose (400 mg) versus high-dose (800 mg) Imatinib therapy showed the latter was associated with a longer time to disease progression but did not improve overall survival with slightly improved progression-free survival [1, 10]. However, a higher dose of imatinib was also associated with a much higher rate of side effects. Side effects of imatinib therapy include edema, muscle cramps, nausea, vomiting, fatigue, and rash. Hematologic effects include anemia, neutropenia, and elevated liver function tests [1, 9].

Sunitinib, an inhibitor of KIT, PDGFRs, VEGFT 1, 23, FLT3, and RET, was approved as a second-line therapy for advance GISTs after imatinib resistance and/or tolerance [4, 10, 64]. Sunitinib scheduled dosing consists of 50 mg per day for four weeks followed by a two-week rest period. Sunitinib potentially inhibits double mutation of the ATP binding pocket which is not possible with imatinib, but has little activity against double mutation in the activation loop, making it more potent against imatinib-resistant ATP binding pocket mutation but inferior potency against the activation loop [10]. Side effects of sunitinib include fatigue, diarrhea, skin discoloration, nausea, dysgeusia, stomatitis, vomiting, hand foot syndrome, dyspepsia, dry mouth, and glossodynia [65]. Most frequent hematologic side effects in decreasing order of frequency include leukopenia, neutropenia, anemia, and thrombocytopenia [64].

#### 7.2.1. Postoperative Imatinib

Interim results from ACOSOG Z9001 phase III double-blind trial for KIT-positive GIST showed improvement of RFS with imatinib treatment postoperatively. ASCOG Z9001 stratified risk based only on tumor size [5, 66]. Another study by de Matteo et al. on 713 patients who completed one year of postoperative imatinib treatment showed a significant improvement of relapse free survival (RFS) but not in overall survival (OS) [1, 5]. Two large trials in Europe are investigating RFS in postoperative imatinib treatment: the phase III trial EORTC/GSF/GEIS/AGIT 62024 and the phase III randomized, multicenter study SSGXVIII/AIO [5, 66].

Postoperative imatinib treatment is recommended if the tumor is removed grossly, but the operative specimen has positive microscopic margins, designated as R1 resection, or if a gross visible tumor was left behind designated as R2 resection. Observation is all that is recommended if an R0 resection (negative microscopic margins) was achieved [5, 63]. The consensus at this time is to treat patient in a multidisciplinary approach based on biopsy margin, tumor size, mitotic rate, site, immunohistochemical staining, and mutational status [5, 63, 66, 67] (Figure 3).

#### 7.2.2. Imatinib Resistance

Most GIST patients will achieve the clinical benefits with imatinib, but an estimated 10% will progress within 3 to 6 months of initiating therapy. Such cases are described as showing primary resistance to treatment. Another 40% to 50% of patients will go on to develop resistance within the first two years [10]. In the cases reviewed, 1 out of 5 GISTs in the stomach and the small intestine developed resistance/relapse to imatinib treatment within two years (Table 2).

Primary imatinib resistance is observed in roughly 10% of all genotypic subtypes of GIST. Most cases that show primary resistance are *kit* and PDGFRA wild type, those with *kit *exon 9 mutations and those with PDGFRA D824V  mutation. Imatinib only binds to the inactive form of PDGFRA. Furthermore, the D824V mutation of PDGFRA results in change in the kinase activation loop which favors active conformation, thereby making it resistant to imatinib. In patients who do not harbor the PDGFRA or *kit* mutation, the mechanism of resistance is potentially a mutation in another alternate signaling pathway [10].

Delayed imatinib resistance is most often associated with expression of tumor clones with secondary *kit *or PDGFRA mutations. In phase II clinical trial of imatinib, 67% of patients with delayed resistance had tumor clones with one or more secondary kinase mutation. All secondary *kit *and PDGFRA mutations were found on GIST with underlying primary *kit* and primary PDGFRA mutation, respectively. No secondary mutations were noted in samples after imatinib that lacked a primary mutation, such as wild-type GISTs. *Kit* mutation also shows mutational heterogeneity; a biopsy of one progressing lesion may not be a representative of others. Hence, making genotyping for resistance is more difficult and is not recommended for routine clinical management [10].

#### 7.2.3. Sunitinib Resistance

The response to sunitinib correlates closely with the tumor mutation status prior to imatinib treatment. The median progression-free survival and overall survival with sunitinib were significantly longer for patients with secondary *kit *mutations in exon 13 or 14 than those with secondary *kit* mutations in exon 17 or 18. This correlates that sunitinib potentially inhibits the phosphorylation of KIT double mutation in ATP binding site but not in mutations of the activating loop. Sunitinib also has increased potency against imatinib-resistant ATP binding pocket mutation but inferior potency against the activation loop [10]. No case report of sunitinib resistance was reported in our review (Table 1, Figure 2). 

## 8. Future Direction

### 8.1. Monoclonal Antibodies

Newer monoclonal antibodies are being developed for treatment of imitinib/sunitinib resistance GISTs. These include nilotinib, sorafenib, dovitinib, crenolanib, pazopanib, and dasatinib.

Nilotinib (Tasigna) is an orally bioavailable aminopyrimidine-derivative Bcr-Abl tyrosine kinase inhibitor with antineoplastic activity [68]. It is designed to overcome imatinib resistance and is currently approved by the FDA for the treatment of chronic lymphocytic leukemia. Preliminary studies with nilotinib have shown that it can provide a clinical benefit in patients who have failed first- and second-line therapies by binding to KIT and PGDFRA. It is well tolerated in patients with advanced GIST [68, 69]. Phase II trials are underway to assess its efficacy as third-line therapy. The preliminary results from a recent phase III trial to investigate the efficacy of nilotinib as first-line treatments in patients without prior imatinib therapy are unlikely to demonstrate superiority over the standard of care, which is imatinib, hence it was discontinued [67].

Dasatinib is structurally unrelated to imatinib, possibly demonstrating a higher affinity to KIT. It inhibits KIT autophosphorylation and KIT-dependent activation of downstream pathways. Preclinical cell studies indicate that dasatinib may inhibit the KIT D816V mutation that is resistant to imatinib [70]. A study by Schittenhelm et al. also indicates a possible activity against KIT activation loop mutations D816Y, D116F and D816V making it useful for imatinib-resistant GISTs [70, 71]. A multicenter phase II trial sponsored by the Swiss Group for clinical research is testing dasatinib as a first-line treatment in gastrointestinal stromal tumors (http://www.clinicaltrials.gov/).

Crenolanib (CP-868,596) developed by AROG Pharmaceuticals is an orally bioavailable small molecule targeting the platelet-derived growth factor receptor (PDGFR), with potential antineoplastic activity. Phase I and phase IB trials are assessing its safety, tolerability, and pharmacokinetics when combined with other drugs and chemotherapeutic agents. Both trials demonstrated well tolerability with promising results [72, 73]. Crenolanib is undergoing phase II trials for the treatment of GISTs with PDGFRA mutation, which are most likely resistant to imatinib and sunitinib [74].

Pazopanib (Votrient) is a small-molecule inhibitor of multiple protein tyrosine kinases with potential antineoplastic activity. Pazopanib selectively inhibits vascular endothelial growth factor receptors (VEGFR)-1, -2, and -3, KIT, and platelet derived growth factor receptor (PDGFR), which inhibit angiogenesis in tumors were these receptors are bound [75]. Pazopanib is FDA approved for renal cell carcinoma treatment. It is undergoing clinical trial for treatment of advanced solid tumors, including GISTs (http://www.clinicaltrials.gov/).

Dovitinib (TKI258) is another KIT/PDGFRA inhibitor and VEGF inhibitor developed by Novartis. Initial phase I studies demonstrated well tolerability in 35 patients. Its activity against the tyrosine kinase postulated its possible efficacy against other solid tumors such as GIST. The most common side effects with dovitinib include fatigue, nausea, vomiting, and diarrhea [76]. A phase II trial is on its way as a third-line treatment for imitinib/sunitinib-resistant GIST (http://www.clinicaltrials.gov/).

Sorafenib (BAY 43-9006, Nexavar) is an oral multi-kinase inhibitor that blocks the RAF kinase and VEGF receptors 2 and 3 to target tumor cell growth and angiogenesis. It also blocks PDGFR-B, KIT, FLT-3, and RET [77, 78]. Sorafenib was initially approved by the FDA for the treatment of kidney cancer. Sorafenib is undergoing phase II trial as fourth-line treatment in imatinib-, sunitinib-, and nilotinib-resistant metastatic GIST (http://www.clinicaltrials.gov/).

### 8.2. HSP-90

Heat shock protein 90 (Hsp90) is an ATP-dependent chaperone protein required for the proper folding and activation of other cellular proteins, particularly kinases [79–81]. Hsp-90 interacts with more than 200 proteins; many of these “client proteins” include AKT, BCR-ABL, NPM-ALK, BRAF, KIT, MET, EGFR, FLT3, HER2, PDGFRA, VEGFR, which are expressed in CML, CLL, lymphoma, AML, non-small-cell lung cancer, breast cancer, prostate cancer, and GIST [80, 81]. It has been shown to be critical to cancer cell growth, proliferation, and survival. They are the new targets of clinically validated cancer drugs.

HSP-90 has a critical role in the maintenance of multiple oncogenic pathways and is required to maintain the proper folding, the stability, and the functionally active conformation of many aberrant oncoproteins [81]. Pharmacologic inhibition of HSP-90 by small molecules destabilizes the cancer cell protein leading to degradation by proteasomal enzymes [80].

The first Hsp90 inhibitor to enter clinical trials was the geldanamycin (GM) derivative 17-allylamino-17-demethoxygeldanamycin (17-AAG). HSP-90 inhibitors include the two 17-AAG formulations, tanespimycin and IPI-504. Synthetic HSP-90 inhibitors are also being developed, which includes purine-scaffold Hsp90 inhibitor CNF2024/BIIB021, the isoxazole derivative VER-52296/NVP-AUY922, and carbazol-4-one benzamide derivative SNX-5422 [80]. A third type of Hsp90 is being developed by Synta Pharmaceuticals, the STA 9090. It is an HSP-90 inhibitor unrelated to the ansamycin family and is undergoing phase II clinical trial for patients with GISTs [81]. Two phase II trials are underway for AUY-933, the isoxazole derivative of 17-AAG in treatment for refractory GISTs [80].

STA-9090 (ganetespib) is a novel second-generation, resorcinol-containing triazole heat shock protein inhibitor (HSP-90) that has shown the ability to inhibit multiple kinases with comparable potency to, and a broader activity profile than, specific kinase inhibitors such as imatinib, erlotinib, and sunitinib in preclinical trials. STA-9090 binds to the ATP-binding pocket at the N-terminus of Hsp90 and acts as a potent Hsp90 inhibitor. STA-9090 has shown potency 10 to 100 times greater than the geldanamycin family of Hsp90 inhibitors, as well as activity against a wider range of kinases. In vivo models have shown strong efficacy in a wide range of cancer types, including cancers resistant to Gleevec, Tarceva, and Sutent [81, 82]. Phase II trials are underway to determine its effectiveness in the treatment of patients with metastatic and/or unresectable tumor that received prior imatinib or sunitinib treatment [81].

## 9. Conclusion

GIST is a tumor with growing concern. Despite surgery and neoadjuvant treatment, it remains a source of resistance with a devastating impact on mortality and healthcare. The diagnosis of GIST is often delayed owing to its indolent symptoms that only present in advance and sometimes unresectable stage. Immunohistochemical staining is a useful aid in diagnosing GISTs. Newer staining techniques, such as the highly specific DOG1, sound promising in diagnosing GIST and eventually would channel patients to its proper treatment. AFIP is still the most commonly used risk stratification for prognosis and treatment, although its complexity has raised questions on its usefulness. Newer methods of staging using TNM system is available but needs further validation on its role in predicting prognosis and treatment outcome.

 With the understanding of the molecular biology on how GIST progresses together with the advancement of immunohistochemical staining, newer drugs are being developed that specifically target areas were tyrosine kinase and PDGFRA are being activated. It has also revolutionized our understanding of drug resistance and how to overcome such. Surgery still remains as the primary mode of treatment despite a high incidence of recurrence, owing to the lack of alternative treatment options. Improvement in surgical techniques has decreased the incidence of tumor recurrence from tumor seeding. Postoperative imatinib treatment has also shown to improve relapse-free survival (RFS) but not overall survival (OS) and needs further studies which, at present, are being done by 2 large clinical trials in Europe. With the occurrence of imatinib and sunitinib resistance drugs, third- and fourth-generation tyrosine kinase and PDGFRA inhibitors are being developed and undergoing clinical trial that would hopefully change the course of management of GISTs in the very near future. 

## Figures and Tables

**Table 1:** Review of case reports (*n* = 32).

Age/sex/history	Published/author	Location of GISTs	Primary symptom	Tumor size (cm)	Primary treatment	Immunohistochemistry/mutational analysis	Pathology	Postop. imatinib/sunitinib treatment	Relapse
25 y.o./F	1/2009/Scherjon et al. [17]	Duodenum	Palpable mass	17 × 16 × 5; 13 × 8 × 3.5	Surgical	(+) CD117/*c-kit*; weakly (+) MS-actin and SM-actin	Spindle cells	No	Yes, 6 years after surgery
74 y.o./F	1/2009/Terada [8]	Uterus	Pelvic pain	13 × 15 × 12	Surgical	(+) CD117/*c-kit*; weakly (+) PDGFRA	Spindle cell	Yes (I)	No, 2 years after treatment
52 y.o./F	11/2009/Trabelsi et al. [18]	Pancreas	Epigastric pain	10.5 × 7 × 3	Surgical	(+) CD117/*c-kit*; (+) CD34	Mixed Spindle-epithelioid cells	No	No, 10 months after surgery
58 y.o./F	1/2008/Papaspyros and Papagiannopoulos [19]	Esophagus	Pleuritic chest pain	10 in dia.	Surgical	(+) CD117/*c-kit*; (+) CD34	Spindle-shaped cells; >5/50 hpf	Not reported	Not reported
65 y.o./F	10/2005/Lin et al. [20]	Gastric corpus	Incidental finding	1.1 × 0.7 × 0.3	Surgical	(+) CD117/*c-kit*; (+) CD34; (+) NSE; (+) S-100	Not reported	No	No, 28 mos. after surgery
66 y.o./M	8/2007/Efstathios et al. [21]	Small bowel	Small bowel obstruction	10 in dia	Surgical	(+) CD117/*c-kit*; (+) SMA, (+) S-100	Spindle-shaped cells; 8–10/hpf	Yes (I)	Yes, 2 years after treatment
60 y.o./F/NF-1	2010/Ohtake et al. [22]	Multiple jejenum/duodenum	Incidental finding	1.4 dia.	Surgical	(+) CD117/*c-kit*; (+) CD34	Spindle-shaped cells/<5/50/hpf	No	No, 2 years after surgery
34 y.o./F	5/2009/Masoodi et al. [23]	Transverse colon	Abdominal lump	8.0 in dia.	Surgical/conventional chemotherapy	(+) CD117/*c-kit *	Spindle shaped/no mitotic activity	No	No, 8 months after treatment
75 y.o./M	5/2008/Laperouse et al. [9]	Body of stomach	GI bleed	5.0 × 3.5 × 3.5	Surgery	(+) CD117/*c-kit*; (+) CD34, (+) desmin	Spindle-shaped cells; <5/50/hpf	Yes (I)	Not reported
68 y.o./M	10/2004/Demetri et al. [7]	Retroperitoneum contiguous with post. wall stomach, pancreas, multiple liver mass	Incidental finding	13 in dia.	Surgery	(+) CD117/*c-kit*; (+) CD34	Spindle-shaped cells/low mitotic rate	Yes (I) initially then (S)	Not reported
54 y.o./M	02/2010/Freeman et al. [24]	Greater curvature of stomach	Upper Abdominal pain	1.3 on dia.	Surgery	(+) CD117/*c-kit *	10/hpf	Yes (I)	No, 18 months after treatment
42 y.o./F	4/2007/Pinto et al. [25]	Small bowel	Lower abdominal pain	4.8 in dia	Surgery	(+) CD117/*c-kit*; (+) CD34	Spindle-shaped cell; 9/50 hpf	Yes (I)	No, 16 months after treatment
76 y.o./F	4/2007/Pinto et al. [25]	Stomach	Dysuria, altered bowel movement, abdominal discomfort	17 in dia.	Surgery	(+) CD117/*c-kit*; (+) CD34	Spindle-shaped cell; 7/50 hpf mitotic index	No	No, 12 months after treatments
61 y.o./F	6/2011/Fan et al. [26]	Rectum	Lower GI bleed	8.0 in dia	Surgery	(+) CD117/*c-kit*; (+) CD34; (+) SMA	5/50 hpf mitotic index	No	Not reported
17 y.o./M	08/2009/Luo et al. [27]	R liver lobe	Incidental finding	5.1 × 3.8 × 4.6	Radiofrequency ablation	(+) CD117/*c-kit*; (+) CD34; (+) vimentin	Spindle cells/no mitosis	No	No, 3 mos. after treatment
28 y.o./M	07/2010/Yucel et al. [28]	Duodenum	Abdominal pain	24 in dia.	Surgery	(+) CD117/*c-kit*; (+) CD34	0/50 hpf mitotic index	No	Not reported
62 y.o./M	07/2010/Yucel et al. [28]	Fundus of stomach	Abdominal pain	10 in dia	Surgery	(+) CD117/*c-kit*; (+) CD34	<5/50 hpf mitotic index	No	Not reported
38 y.o./M	07/2010/Yucel et al. [28]	Jejunum	Abdominal pain, rectal bleeding	2.5 in dia.	Surgery	(+) CD117/*c-kit*; (+) CD34	<1/50 hpf mitotic index	No	Not reported
62 y.o./M	01/2009/Lee et al. [29]	Stomach	Symptomatic anemia	Not reported	Surgery	(+) CD117/*c-kit *	Spindle-shaped cells; 60/50 hpf	Yes (I)	Yes, Increase in tumor size, liver 2 years after therapy
80 y.o./M	2011/Theodosopoulos et al. [30]	Stomach	Epigastric discomfort, nausea, weight loss	3.0 in dia.	Surgery	(+) CD117/*c-kit *	Severe nuclear atypia; 6/50 hpf mitotic index	Yes (I)	No, 1 year after treatment
52 y.o./F	12/2009/Ulusan et al. [31]	Small intestine	Abdominal pain	18 × 13 × 7	Surgery	(+) CD117/*c-kit*; weakly (+) S-100, SMA, CD34	3/50 hpf mitotic index	Yes (I)	Not reported
31 y.o./F	06/2003/Sakamoto et al. [32]	Duodenum/pancreas	Not mentioned	Not reported	Surgery	(+) CD117/*c-kit *	Low-grade malignant potential	No	Not reported
28 y.o./F	2010/Abdel-Ghaffar [33]	Mesentery	Abdominal pain, distention	20 × 18 × 16	Surgery	(+) CD117/*c-kit*; (+) CD34	Spindle-shaped cells with twisted nuclei; 7/50 hpf	No	No, 3 years after treatment
65 y.o./M	06/2007/Todoroki et al. [34]	Greater omentum	Abdominal mass	20 × 17 × 6	Surgery	(+) CD34; weakly (+) CD117/*c-kit*; PDGFA gene mutation on exon 12	Mixed spindle-epitheloid; 2/50 hpf	Yes	No, 6 months after treatment
38 y.o./M	06/2011/Dhull et al. [35]	Jejunum	Abdominal pain/abdominal fullness	10 × 15	Surgery	(+) CD117/*c-kit *	Spindle-shaped cells; high grade stromal tumor	Yes (I)	No, 6 months after therapy
65 y.o./F	3/2008/Dickhoff et al. [36]	Rectum	Abdominal pain	15 in dia.	Surgery	(+) CD117/*c-kit *	High mitotic rate	No	Yes, 1 year after
62 y.o./M	3/2008/Dickhoff et al. [36]	Rectum, metastatic	Obstipation, voiding problem	8.9 × 8.6	Imatinib	(+) CD117/*c-kit *	High mitotic rate	N/A	No, regression of tumor on ff up
55 y.o./M/NF-1	11/2009/Takeuchi et al. [37]	Duodenum	Incidental finding	2.5 × 2.5 × 2.3	Surgery	(+) CD117 *c-kit*/(+) CD34,	Spindle-shaped cells; 3/50 hpf	No	No, after 2 years
56 y.o./F	3/2010/Tahara et al. [38]	Lesser curvature of gastric corpus	Abdominal pain, nausea, anemia	1.8 × 1.5 × 1.0	Surgery	(+) CD117 *c-kit*/(+) CD34	Spindle-shaped cells; 0/50 hpf	No	No, after 18 mos.
66 y.o./F/NF-1	10/2010/Kitagawa et al. [39]	Jejunum	Partial seizures	6.0 in dia.	Surgery	(+) CD117/*c-kit*/wild type KIT	Spindle-shaped cells; 5/50 hpf	No	Not reported
70 y.o./M/NF-1	5/2011/Takakura et al. [40]	Jejunum	Melena, fatigue	2.8 × 2.4 × 1.8	Surgery	(+) CD117/*c-kit*/(+) CD34	Spindle-shaped cell; low mitotic activity	No	Not reported
28 y.o./F	2010/Varras et al. [41]	Small intestine	Abdominal pain	13 × 10 × 9.0	Surgery	(+) CD117/*c-kit*/(+) CD34	Mixed spindle-epithelioid; 5/50 hpf	Yes (I)	No, 3 years after

Abbreviations**: **I—imatinib. S—sunitinib. hpf—high-power field. NF-1—neurofibromatosis type 1. SMA—smooth muscle actin. NSE—nonspecific enolase. PDGFRA—platelet-derived growth factor receptor alpha.

**Table 2:** AFIP-Miettinen risk stratification system.

Risk stratification of primary GIST by mitotic index, size, and site*					
Tumor parameters		Risk of progressive disease* (%)			
Mitotic index	Size	Gastric	Duodenum	Jejunum/ileum	Rectum
≤5 per 50 hpf	≤2 cm**	None (0%)	None (0%)	None (0%)	None (0%)
	>2 ≤ 5 cm	Very low (1.9%)	Low (4.3%)	Low (8.3%)	Low (8.5%)
	>5 ≤ 10 cm	Low (3.6%)	Moderate (24%)	(Insuff. data)	(Insuff. data)
	>10 cm	Moderate (10%)	High (52%)	High (34%)	High (57%)

	≤2 cm	None^†^	High^†^	(Insuff. data)	High (54%)
>5 per 50 hpf	>2 cm ≤ 5 cm	Moderate (16%)	High (73%)	High (50%)	High (52%)
	>5 cm ≤ 10 cm	High (55%)	High (85%)	(Insuff. data)	(Insuff. data)
	>10 cm	High (86%)	High (90%)	High (86%)	High (71%)

These Data are based on long-term followup of 1055 gastric, 629 small intestinal, 144 duodenal, and 111 rectal GISTs.

Abbreviations: GIST—gastrointestinal stromal tumor; hpf—high power field. Insuff—insufficient.

*Defined as metastasis or tumor-related death.

^†^Denotes small numbers of cases.

Adapted from Miettinen and Lasota, 2006 with permission from Elsevier [48].

**Table 3:** The new TNM risk stratification system [59].

TNM classification for gastric GIST including primary, solitary omental GISTs				
Stage	T	N	M	Mitotic rate per 50 hpf

Stage IA	T1,T2	N0	M0	<5
Stage IB	T3	N0	M0	<5
Stage II	T1, T2	N0	M0	>5
	T4	N0	M0	<5
Stage IIIA	T3	N0	M0	>5
Stage IIIB	T4	N0	M0	>5
Stage IV	Any T	N1/Any N	M0/M1	Any rate

TNM classification for small intestinal GIST including esophagus, colon, rectum, mesentery, and other less common sites				
Stage	T	N	M	Mitotic rate per 50 hpf

Stage IA	T1,T2	N0	M0	<5
Stage II	T3	N0	M0	<5
Stage IIIA	T1	N0	M0	>5
	T4	N0	M0	<5
Stage IIIB	T2, T3, T4	N0	M0	>5
Stage IV	Any T	N1/any N	M0/M1	Any rate

T-primary tumor				
TX	cannot be assessed			
T0	no evidence of primary tumor			
T1	Tumor ≤2 cm			
T2	Tumor >2 cm to 5 cm			
T3	Tumor >5 cm to 10 cm			
T4	Tumor >10 cm			

N-regional lymph node				
NX	cannot be assessed			
N0	No lymph node involvement			
N1	Lymph node involvement			

M-distant metastasis				
M0	No distant metastasis			
M1	Distant metastasis			
